# Comparison of cerebrospinal fluid, plasma and neuroimaging biomarker utility in Alzheimer’s disease

**DOI:** 10.1093/braincomms/fcae081

**Published:** 2024-03-15

**Authors:** Karin L Meeker, Patrick H Luckett, Nicolas R Barthélemy, Diana A Hobbs, Charles Chen, James Bollinger, Vitaliy Ovod, Shaney Flores, Sarah Keefe, Rachel L Henson, Elizabeth M Herries, Eric McDade, Jason J Hassenstab, Chengjie Xiong, Carlos Cruchaga, Tammie L S Benzinger, David M Holtzman, Suzanne E Schindler, Randall J Bateman, John C Morris, Brian A Gordon, Beau M Ances

**Affiliations:** Department of Neurology, Washington University in St Louis, St Louis, MO 63110, USA; Department of Neurosurgery, Washington University in St Louis, St Louis, MO 63110, USA; Department of Neurology, Washington University in St Louis, St Louis, MO 63110, USA; Department of Radiology, Washington University in St Louis, St Louis, MO 63110, USA; Department of Radiology, Washington University in St Louis, St Louis, MO 63110, USA; Department of Neurology, Washington University in St Louis, St Louis, MO 63110, USA; Department of Neurology, Washington University in St Louis, St Louis, MO 63110, USA; Department of Radiology, Washington University in St Louis, St Louis, MO 63110, USA; Department of Radiology, Washington University in St Louis, St Louis, MO 63110, USA; Department of Neurology, Washington University in St Louis, St Louis, MO 63110, USA; Department of Neurology, Washington University in St Louis, St Louis, MO 63110, USA; Department of Neurology, Washington University in St Louis, St Louis, MO 63110, USA; Department of Neurology, Washington University in St Louis, St Louis, MO 63110, USA; Knight Alzheimer Disease Research Center, Washington University School of Medicine, St Louis, MO 63110, USA; Knight Alzheimer Disease Research Center, Washington University School of Medicine, St Louis, MO 63110, USA; Division of Biostatistics, Washington University in St Louis, St Louis, MO 63110, USA; Knight Alzheimer Disease Research Center, Washington University School of Medicine, St Louis, MO 63110, USA; Department of Psychiatry, Washington University School of Medicine, St Louis, MO 63110, USA; Department of Radiology, Washington University in St Louis, St Louis, MO 63110, USA; Knight Alzheimer Disease Research Center, Washington University School of Medicine, St Louis, MO 63110, USA; Department of Neurology, Washington University in St Louis, St Louis, MO 63110, USA; Knight Alzheimer Disease Research Center, Washington University School of Medicine, St Louis, MO 63110, USA; Department of Neurology, Washington University in St Louis, St Louis, MO 63110, USA; Knight Alzheimer Disease Research Center, Washington University School of Medicine, St Louis, MO 63110, USA; Department of Neurology, Washington University in St Louis, St Louis, MO 63110, USA; Department of Neurology, Washington University in St Louis, St Louis, MO 63110, USA; Knight Alzheimer Disease Research Center, Washington University School of Medicine, St Louis, MO 63110, USA; Department of Radiology, Washington University in St Louis, St Louis, MO 63110, USA; Knight Alzheimer Disease Research Center, Washington University School of Medicine, St Louis, MO 63110, USA; Department of Neurology, Washington University in St Louis, St Louis, MO 63110, USA; Department of Radiology, Washington University in St Louis, St Louis, MO 63110, USA; Knight Alzheimer Disease Research Center, Washington University School of Medicine, St Louis, MO 63110, USA

**Keywords:** Alzheimer’s disease, biomarkers, machine learning

## Abstract

Alzheimer’s disease biomarkers are crucial to understanding disease pathophysiology, aiding accurate diagnosis and identifying target treatments. Although the number of biomarkers continues to grow, the relative utility and uniqueness of each is poorly understood as prior work has typically calculated serial pairwise relationships on only a handful of markers at a time. The present study assessed the cross-sectional relationships among 27 Alzheimer’s disease biomarkers simultaneously and determined their ability to predict meaningful clinical outcomes using machine learning. Data were obtained from 527 community-dwelling volunteers enrolled in studies at the Charles F. and Joanne Knight Alzheimer Disease Research Center at Washington University in St Louis. We used hierarchical clustering to group 27 imaging, CSF and plasma measures of amyloid beta, tau [phosphorylated tau (p-tau), total tau t-tau)], neuronal injury and inflammation drawn from MRI, PET, mass-spectrometry assays and immunoassays. Neuropsychological and genetic measures were also included. Random forest-based feature selection identified the strongest predictors of amyloid PET positivity across the entire cohort. Models also predicted cognitive impairment across the entire cohort and in amyloid PET-positive individuals. Four clusters emerged reflecting: core Alzheimer’s disease pathology (amyloid and tau), neurodegeneration, AT8 antibody-associated phosphorylated tau sites and neuronal dysfunction. In the entire cohort, CSF p-tau181/A*β*40_lumi_ and A*β*42/A*β*40_lumi_ and mass spectrometry measurements for CSF pT217/T217, pT111/T111, pT231/T231 were the strongest predictors of amyloid PET status. Given their ability to denote individuals on an Alzheimer’s disease pathological trajectory, these same markers (CSF pT217/T217, pT111/T111, p-tau/A*β*40_lumi_ and t-tau/A*β*40_lumi_) were largely the best predictors of worse cognition in the entire cohort. When restricting analyses to amyloid-positive individuals, the strongest predictors of impaired cognition were tau PET, CSF t-tau/A*β*40_lumi_, p-tau181/A*β*40_lumi_, CSF pT217/217 and pT205/T205. Non-specific CSF measures of neuronal dysfunction and inflammation were poor predictors of amyloid PET and cognitive status. The current work utilized machine learning to understand the interrelationship structure and utility of a large number of biomarkers. The results demonstrate that, although the number of biomarkers has rapidly expanded, many are interrelated and few strongly predict clinical outcomes. Examining the entire corpus of available biomarkers simultaneously provides a meaningful framework to understand Alzheimer’s disease pathobiological change as well as insight into which biomarkers may be most useful in Alzheimer’s disease clinical practice and trials.

## Teaser

Meeker *et al*. use machine learning methods on 27 plasma, CSF, MRI and PET Alzheimer’s disease biomarkers to demonstrate that although the number of Alzheimer’s disease biomarkers has rapidly expanded, many are interrelated and few strongly predict clinical outcomes.

## Graphical Abstract

**Graphical Abstract:**
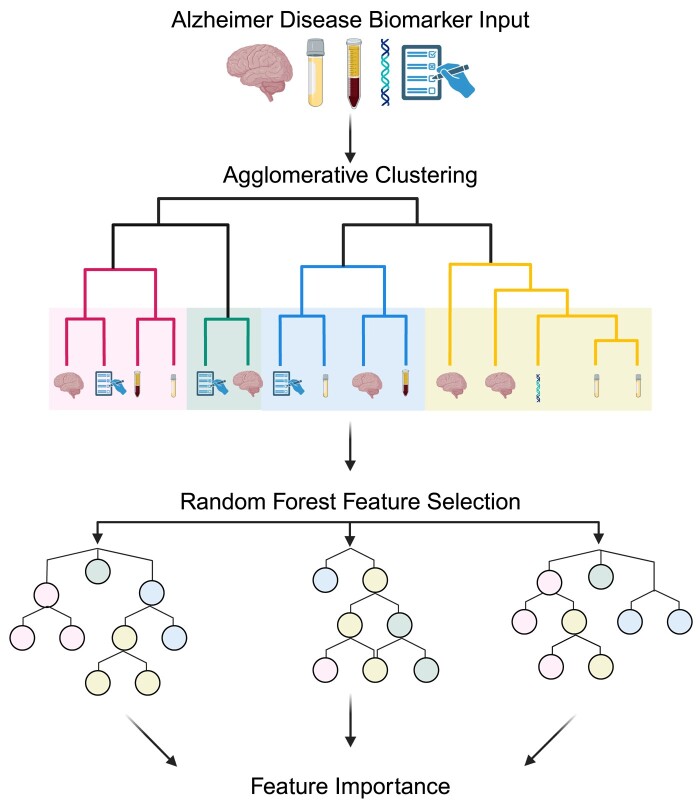

## Introduction

The defining neuropathological features of Alzheimer’s disease include amyloid beta (A*β*) plaques and neurofibrillary tau tangles (NFTs), which are associated with neuronal dysfunction and risk for cognitive decline.^1^ Identifying biomarkers that reflect these pathological hallmarks is important for the early diagnosis of Alzheimer’s disease, tracking progression of the disease and response to treatment. Despite a growing number of biomarkers used in the field, it is largely unknown which provide unique information and have the greatest utility in predicting disease outcomes.

Alzheimer’s disease biomarkers are measured through multiple modalities, including neuroimaging, CSF and blood. Amyloid PET is considered the gold standard for quantifying *in vivo* A*β* deposits in the brain.^2^ Declines in CSF and plasma A*β*42/40 correspond with increasing amyloid PET^3^ and are used as proxies for plaque deposition. Tau was first measured in the CSF using immunoassays to quantify levels of total tau and tau phosphorylated at threonine181 (T181)^4^ and elevated levels were thought to reflect NFTs. Mass spectrometry^5^ and plate-based^6^ approaches have expanded the number of measured phosphorylation sites including T217, T231, T231, T111 and T205.^7,8^ In parallel to the expansion of biofluid measures, PET ligands that bind to NFTs were introduced providing *in vivo* measures of aggregated tau.^9^ Together, a new framework has emerged where levels of, or the occupancy of (i.e. % phosphorylated to non-phosphorylated) CSF pT217, pT181, pT111 and pT231 increase early in the disease course and are associated with amyloid PET, while increases in pT205 are more strongly associated with downstream tau PET, neurodegeneration and the onset of dementia.^10-14^

There has also been a rapid expansion in biofluid markers beyond amyloid and tau to those of neuronal integrity and neuroinflammation, sometimes referred to as ‘emerging’ biomarkers. CSF synaptosomal-associated protein-25 (SNAP-25) and neurogranin (Ng) are pre- and post-synaptic proteins, respectively, which decrease as synaptic density is reduced.^15,16^ CSF and plasma neurofilament light (NfL) chain and CSF visinin-like protein 1 (VILIP-1) are considered markers of neuroaxonal and neuronal injury, respectively.^17,18^ The triggering receptor expressed on myeloid cells 2 (TREM2) is expressed in microglia and is involved in the innate immune response with CSF-soluble TREM2 (sTREM2) reflecting neuroinflammation.^19^ CSF chitinase-3-like protein 1 (YKL-40) has also been used as a biomarker of neuroinflammation.^20^ Notably, these biomarkers are not Alzheimer’s disease specific but instead reflect the extent of neuronal and immunological dysfunction.^21^

While an increase in the number of Alzheimer’s disease biomarkers has led to a richer characterization of the biological mechanisms associated with the disease, it has also introduced complexity. Like most diseases, Alzheimer’s disease involves a dynamic set of pathological changes, which manifests differently from patient to patient. Understanding this complexity requires sophisticated analysis techniques performed on large amounts of heterogeneous data to identify novel patterns and associations.^22^ Traditional analysis techniques (e.g. frequentist statistics) are primarily concerned with data inference achieved by analysing a single explanatory variable while considering all other variables confounders.^23^ This is generally achieved via linear modelling and significance testing. However, as the heterogeneity of the data, dimensionality of the data and the possible association among variables continues to increase, so too must the intricacy of the models used to holistically analyse the data.^24^ One such approach is to employ data-driven machine learning (ML) models. ML methods are well suited to learning from heterogeneous data and have proven to be beneficial in characterizing large numbers of variables without the need for strict *a priori* limitations.^25^ For example, unsupervised clustering is a type of exploratory data analysis that has been used extensively in the medical field for revealing underlying relationships among variables, identifying subpopulations in heterogeneous disorders and understanding selective responses to treatment.^26-28^ Similarly, supervised methods, such as random forest, have been widely used for classification and prediction, as well as discovering the strongest predictors of a given outcome variable (e.g. embedded feature selection).^29^ Thus, ML is an advantageous choice for discerning which Alzheimer’s disease biomarkers provide unique information, to comprehensively understand the mechanisms involved in disease progression, and to make informed decisions on the translation of markers to clinical practice and trials.

The present study assessed the cross-sectional relationships among 27 Alzheimer’s disease biomarkers and their ability to predict meaningful clinical outcomes. To understand the interrelated structure of the biomarkers, and how it changes over the course of the disease, agglomerative hierarchical clustering was performed in the entire sample, individuals with preclinical Alzheimer’s disease (i.e. cognitively normal, amyloid PET positive) and cognitively impaired individuals. To understand the relative predictive utility of different biomarkers, we utilized random forest to predict amyloid PET abnormality as well as cognitive status. By integrating these biomarkers into a unified framework, the current research provides insight into which biomarkers may be most useful in research, clinical practice and therapeutic trials of Alzheimer’s disease.

## Materials and methods

### Participants

Data were obtained from 527 community-dwelling volunteers enrolled in memory and aging studies at the Charles F. and Joanne Knight Alzheimer Disease Research Center (ADRC) at Washington University in St Louis, MO. Fluid biomarker collection and neuroimaging were performed at study entry and repeated every 2–3 years. Individuals who had neuroimaging (PET and MRI), CSF and/or plasma collected within 1 year of clinical assessment were included. Age was based on the date of the clinical assessment. All procedures were approved by the Washington University Institutional Review Board, and each participant provided written informed consent.

### Neuropsychological and clinical assessment

Participants underwent a comprehensive evaluation by an experienced clinician that included a detailed interview of a collateral source, a neurological examination of the participant, the Clinical Dementia Rating® (CDR®),^30^ Clinical Dementia Rating Sum of Boxes (CDR-SB) and the Mini-Mental State Examination (MMSE).^31^ A CDR of 0 indicates that the individual is cognitively unimpaired, while CDR 0.5 and CDR 1 indicate very mild and mild dementia.^30^ Participants were subsequently grouped as either CDR 0 (‘cognitively unimpaired’) or CDR > 0 (‘cognitively impaired’). To ensure the impaired group represented Alzheimer’s disease dementia, they were required to be amyloid PET positive within a year of the clinical assessment (*M* = 113.8 days, SD = 154.8 days). Individuals who had a CDR > 0 and who did not have an abnormal PET scan were excluded from analyses (*n* < 10).

### APOE ε4 status and polygenic risk score

DNA samples were collected at enrolment and genotyped using either an Illumina 610 or Omniexpress chip. *APOE* was genotyped by evaluating rs7412 and rs429358 using established methods.^32^ Participants were classified as either *APOE* ε4-positive (ε4/ε4, ε4/ε3, ε4/ε2) or *APOE* ε4-negative (ε2/ε2, ε2/ε3, ε3/ε3).

For the Alzheimer’s disease polygenic risk score (PRS), weighted scores were calculated by using a logarithm of base 2 transformation on single nucleotide polymorphisms (SNPs), as reported in the International Genomics of Alzheimer’s Project (IGAP) study.^33^ SNPs utilized for the score had either a high genotyping rate (>90%) or were reasonable proxies to the IGAP hits.

### Fluid analysis

CSF and blood were collected in the morning after overnight fasting. CSF was collected via gravity drip as previously described.^34^ Blood was collected into EDTA tubes and processed as previously described.^35^ CSF NfL and YKL-40 (Quidel) were measured with enzyme-linked immunosorbent assays (ELISA).^34,36^ CSF NfL values were skewed and were log transformed. CSF Ng, SNAP-25 and VILIP-1 were measured with microparticle-based immunoassays using single molecule counting (SMC) technology.^36^ CSF A*β*42, A*β*40, p-tau181 (p-tau) and total tau (t-tau) were measured with the automated LUMIPULSE immunoassay platform.^37^ CSF A*β*42/40_lumi_, p-tau/A*β*40_lumi_ and t-tau/A*β*40_lumi_ were computed as the normalization for potential individual differences in CSF production by A*β*40 has been shown to significantly improve performance for predicting both amyloid and tau PET pathology.^38,39^ These specific ratios were chosen as they reflect more ‘pure’ biological processes rather than mixed ones (e.g. p-tau/A*β*42) to aid the interpretation of the hierarchal clustering analyses. Plasma NfL (NfL_plasma)_ was measured with Quanterix NF-light assay and values were log-transformed.^40^ Plasma A*β*42 and A*β*40 were measured by the C2N Diagnostics commercial laboratory with an immunoprecipitation-mass spectrometry assay (St Louis, MO, USA),^35^ and the ratio of plasma A*β*42 to A*β*40 (A*β*42/40_plasma_) was evaluated.

CSF tau phosphorylated and non-phosphorylated peptides were analysed with nano-liquid chromatography–high-resolution mass spectrometry (nanoLC–MS/HRMS) using parallel reaction monitoring with higher energy collisional dissociation (HCD) fragmentation. CSF tau phosphorylation levels were estimated using ratios between MS/HRMS transitions of endogenous unphosphorylated peptides and 15N labelled peptides from protein internal standards. The phosphorylation occupancy (percent phosphorylation at different tau sites) was evaluated using the ratio of the MS/HRMS transitions from CSF phosphorylated peptides and corresponding unphosphorylated peptides (pT111/T111, pT153/T153, pT175/T175, pT181/T181, pS199/S199, pS202/S202, pT205/T205, pS208/S208, pT217/T217, pT231/T231).^10^

### Magnetic resonance imaging

Imaging was performed using either a 3 T Siemens Biograph mMR or TIM Trio (Erlangen, Germany) scanner. Scanning parameters can be found in Supplementary material, Section 1.1. T_1_-weighted scans were segmented with FreeSurfer 5.3. Previous work has identified the temporal (inferior, middle and superior), parietal (inferior and superior), entorhinal cortex, precuneus and hippocampus as regions that demonstrate the earliest signs of atrophy and are most affected by Alzheimer’s disease pathology.^41^ An Alzheimer’s disease cortical signature was obtained from thickness values from the left and right hemispheres.

### Positron emission tomography imaging

The amyloid burden was determined using PET [^11^C] Pittsburgh compound B (PiB) or florbetapir (^18^F-AV-45). Data from 30 to 60 min for PiB or 50 to 70 min for ^18^F-AV-45 post-injection window were converted to standard uptake value ratios (SUVR) with the cerebellar cortex serving as the reference region and partial volume corrected using a geometric transfer matrix approach.^42^ SUVRs from the lateral orbitofrontal, medial orbitofrontal, middle temporal, precuneus, rostral middle frontal, superior frontal and superior temporal cortices (defined by FreeSurfer) were averaged to define the mean cortical amyloid SUVR converted to Centiloid to standardize across PiB and ^18^F-AV-45.^43,44^ Amyloid positivity was subsequently defined as having a Centiloid value of 16.4 or greater.^44^

PET tau imaging was performed using [^18^F]-Flortaucipir (AV1451) with the same parameters as amyloid PET. SUVRs were calculated for the 80–100 min post-injection window using the cerebellar grey as a reference region again using a partial volume correction. A summary measure of PET tauopathy, previously defined as the mean of the amygdala, entorhinal cortex, inferior temporal region and lateral occipital regions, was calculated for each participant.^45^ Additional PET details can be found in Supplementary material, Section 1.2.

### Statistical analyses and ML

**Figure 1:**
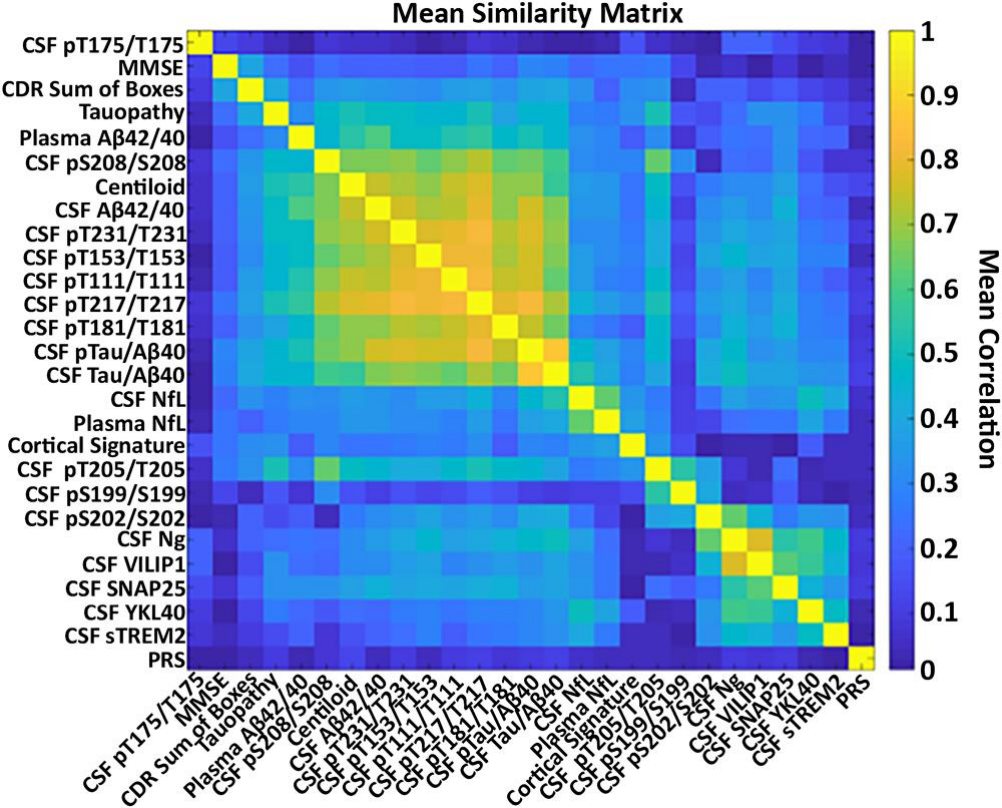
**Similarity matrix used for hierarchical clustering in the entire cohort.** The similarity matrix used for clustering was based on the absolute value of the Spearman correlation between biomarkers. Markers that are more similar are shown in warm colours and markers that are less similar are shown in cooler colours. CDR, Clinical Dementia Rating Scale; MMSE, Mini-Mental State Examination; NfL, neurofilament light; Ng, neurogranin; PRS, polygenic risk score; SNAP-25, synaptosomal-associated protein-25; sTREM2, soluble triggering receptor expressed on myeloid cells 2; VILIP-1, visinin-like protein 1; YKL-40, chitinase-3-like protein 1.

**Figure 2:**
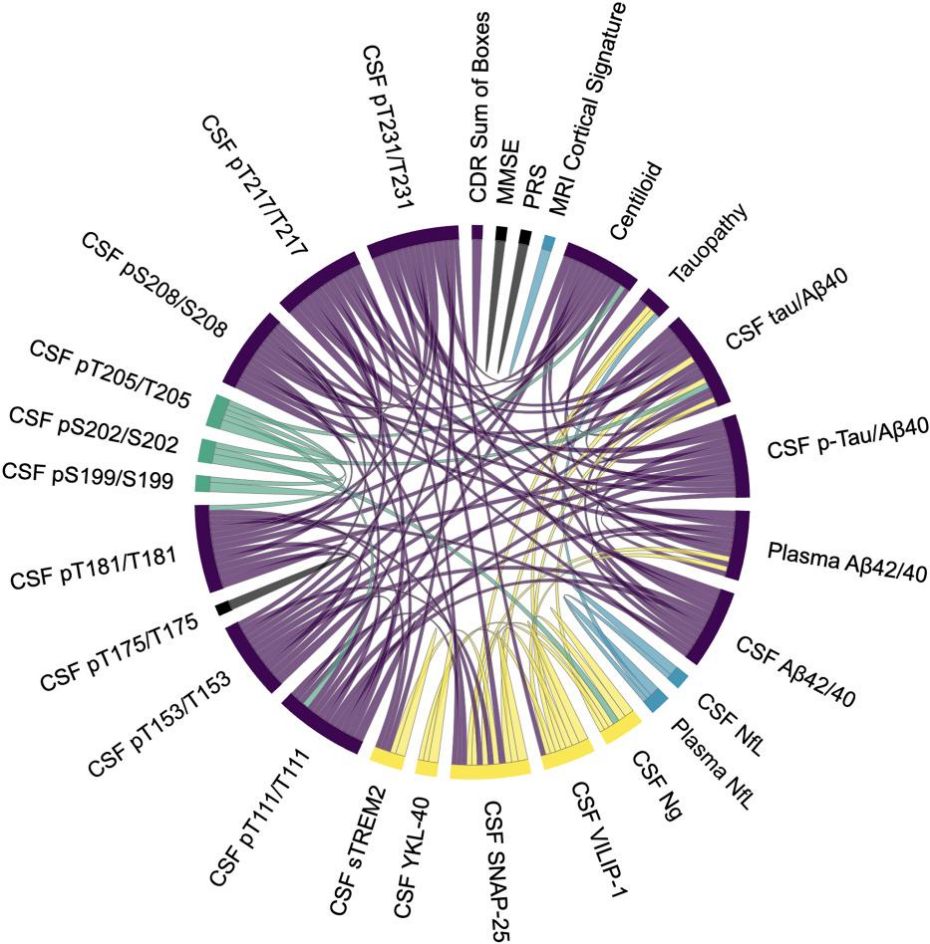
**Circle plot representing Spearman correlations of >0.5 between biomarker pairs across all individuals.** See Supplementary Fig. 8 for a ShinyApp of plots by threshold and group. CDR, Clinical Dementia Rating Scale; MMSE, Mini-Mental State Examination; NfL, neurofilament light; Ng, neurogranin; PRS, polygenic risk score; SNAP-25, synaptosomal-associated protein-25; sTREM2, soluble triggering receptor expressed on myeloid cells 2; VILIP-1, visinin-like protein 1; YKL-40, chitinase-3-like protein 1.

Analyses and figure generation were performed in MATLAB R2021a (Statistics and Machine Learning Toolbox) and the ShinyApp was created in RStudio (version 2022.02.3 + 492) using the ‘shiny’ package. The graphical abstract was created with BioRender.com. Demographic variables were compared by group using χ^2^ tests and ANOVAs. All continuous measures were *z*-scored. Clustering was performed on 27 biomarkers (see Supplementary Table 1 for full list) using agglomerative hierarchical clustering. Hierarchical clustering is a bottom-up approach, where each observation starts in its own cluster, and clusters are merged as they move up the cluster tree.^46^ This approach aids in the interpretability of results, which, due to the hierarchical nature, can be viewed at different scales. The distance metric utilized for clustering was the absolute value of the pairwise Spearman correlation between biomarkers (see Fig. 1 for the similarity matrix and Fig. 2 for associations >0.5). Because the majority of participants were missing at least one biomarker, each correlation was calculated pairwise. Thus, a sample was only removed from the calculation if the missing value was contained in the two biomarkers in question. Individuals missing more than 50% of the biomarkers were excluded. This led to individual correlations being calculated over a variable number of samples, but ensured the maximum number of samples for each correlation calculation was utilized (see Supplementary Figs 1–4 for pairwise counts by group). The Weighted Pair Group Method with Arithmetic Mean^47^ was used for linkage criteria. The optimal number of clusters was identified using the elbow method^48^ based on principal component analysis (see Supplementary Fig. 5) averaged over 1000 permutations, each with 20% random holdout (see Supplementary Fig. 6).

Predictive features for amyloid and cognitive status were ranked according to importance using the embedded feature selection^49^ via random forest.^50^ Amyloid PET and the CDR/MMSE were left out of the amyloid and cognitive status models, respectively, to prevent circularity. Random forests are ensemble methods composed of numerous decision trees, each trained on a random subset of the data. Model results are based on the consensus of all the decision trees.^50^ All models were trained with 10-fold cross-validation (CV).^51^ Within each CV fold, we optimized model hyperparameters (aggregation method, number of learning cycles, learn rate, minimum leaf size, maximum number of splits) using Bayesian optimization.^52^ This optimization process involved 300 iterations, and during each iteration, a separate internal 10-fold CV was performed to robustly estimate the generalization performance of the given hyperparameters. Because each fold was optimized independently, hyperparameters varied by fold. Within each forest, decision trees utilized a curvature test^53^ to construct trees which minimized the *P*-value of the χ^2^ tests of independence between each predictor and the response, as well as each pair of predictors and response. Within each node of the tree, surrogate splits were used to handle missing values. If all surrogate predictor values for an instance were missing, that value was ignored. After training, pruning was used to eliminate unnecessary branches and surrogates. Feature weights, also referred to as predictor strengths here, were averaged from the top performing model from each CV fold (sensitivity and specificity ≥80%) to derive the final feature weights. Feature weights were then rescaled to a [0, 1] interval. Any feature <0.5 was considered a weak predictor.

## Results

### Demographics

The average age of the entire cohort was 70.96 years (SD = 7.02), 53% were female and 17% were cognitively impaired. The cognitively normal (CDR 0) and amyloid PET negative (CN(A−)) group was younger than the preclinical Alzheimer’s disease (cognitively normal, amyloid PET positive [CN(A+)]) group and cognitively impaired (CI; CDR > 0 A+) individuals (*P* < 0.05). CN(A+) and CI groups did not significantly differ in age (*P* > 0.05). CN(A+) and CI groups had a greater proportion of *APOE* ε4 carriers compared to the CN(A−) group (*P* < 0.05). The groups did not significantly differ by sex, race or education (*P* > 0.05). See Table 1 for additional details.

**Table 1:** Participant demographics

	CN(A−)	CN(A+)	CI	*P*
*n*	211	228	88	
Age (years) ± SD	69.72 (7.61)	71.67 (6.53)	72.11 (6.39)	0.003
Sex (%female)	111 (52.6%)	126 (55.3%)	43 (48.9%)	0.582
Race (% NHW)	190 (90.0%)	209 (91.7%)	82 (93.2%)	0.656
*APOE* ε4 carrier (% carrier)	52 (24.8%)	94 (41.4%)	50 (57.5%)	<0.001
Education (years) ± SD	15.97 (2.57)	15.92 (2.32)	15.81 (2.69)	0.873

Cognitively impaired (CI) individuals included Clinical Dementia Rating Scale (CDR) = 0.5 (*n* = 72) and CDR = 1 (*n* = 16). Amyloid positivity was defined as having a PET Centiloid value of 16.4 or greater. APOE ε4, apolipoprotein E; CI, cognitively impaired; CN(A−), cognitively normal, amyloid PET negative; CN(A+), cognitively normal, amyloid PET positive.

### Clustering

**Figure 3:**
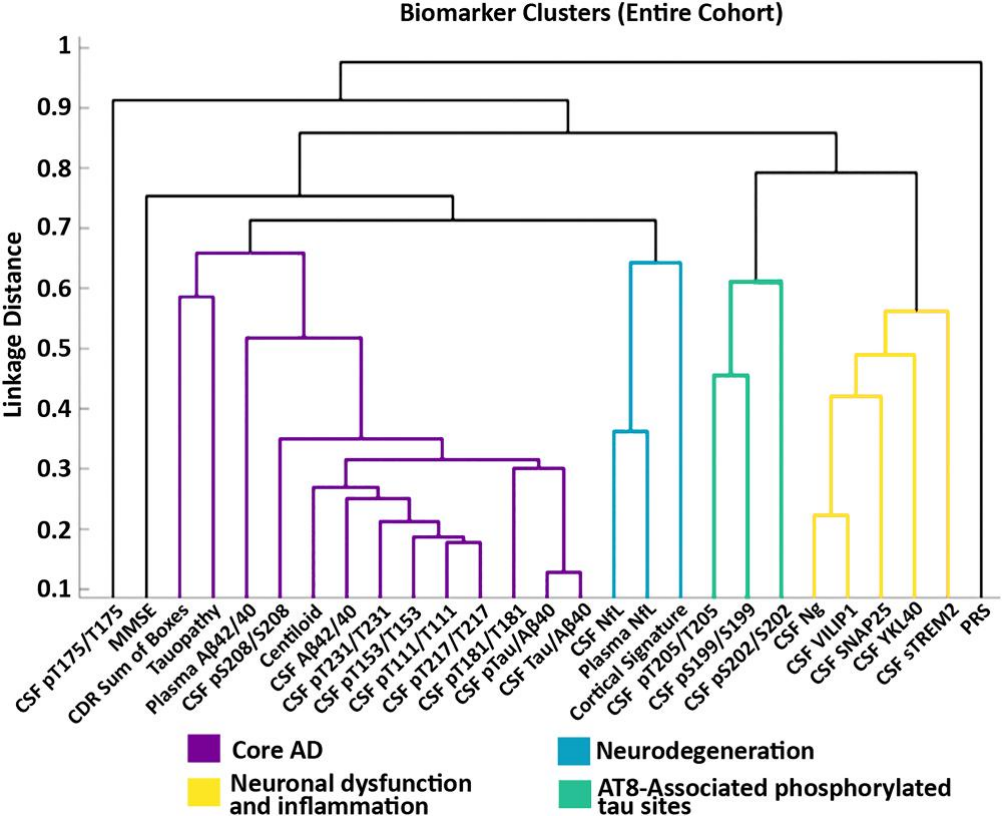
**Agglomerative hierarchical clustering yields four primary clusters for the entire cohort (*n* = 527).** The dendrogram from the clustering analysis identified four main clusters including core Alzheimer’s disease pathology, neurodegeneration, AT8-associated phosphorylated tau sites and neuronal dysfunction and inflammation clusters. Biomarkers from the core Alzheimer’s disease pathology cluster largely demonstrated strong associations with other makers within the cluster although some associations between neuronal dysfunction and inflammation and core Alzheimer’s disease markers were observed. The *y*-axis represents the linkage distance and reflects the absolute value of the pairwise Spearman correlation between biomarkers. Lines are colour coded by cluster. CDR, Clinical Dementia Rating Scale; MMSE, Mini-Mental State Examination; NfL, neurofilament light; Ng, neurogranin; PRS, polygenic risk score; SNAP-25, synaptosomal-associated protein-25; sTREM2, soluble triggering receptor expressed on myeloid cells 2; VILIP-1, visinin-like protein 1; YKL-40, chitinase-3-like protein 1.

Four primary clusters were identified when examining the entire cohort, and consisted of core Alzheimer’s disease pathology, neurodegeneration, AT8-associated phosphorylated tau sites and neuronal dysfunction and inflammation (Fig. 3). The core Alzheimer’s disease pathology cluster was comprised mainly of markers of amyloid and tau and included amyloid PET (Centiloids), A*β*42/40_plasma_, CSF A*β*42/40_lumi_, CSF p-tau/A*β*40_lumi_, CSF t-tau/A*β*40_lumi_, CSF pT217/T217, CSF pT231/T231, CSF pT181/T181, CSF pT111/T111, CSF pT153/T153, CSF pS208/S208, tau PET and CDR-SB. All measures within the core Alzheimer’s disease pathology cluster were strongly interrelated (see Fig. 1 for the Spearman correlations between biomarkers and Fig. 2 for *ρ* of >0.5). Clustering results with concentrations of single analytes (i.e. unnormalized CSF measures of A*β*42_lumi_, A*β*40_lumi_, p-tau_lumi_ and t-tau_lumi_) in addition to the ratios are shown in Supplementary Fig. 7. The neurodegeneration cluster was composed of downstream markers including CSF NfL and NfL_plasma_ and the MRI Alzheimer’s disease cortical thickness signature. The tau-specific AT8 antibody cluster consisted of CSF tau phosphorylation occupancy at sites pT205/T205, pS199/S199 and pS202/S202. Lastly, the neuronal dysfunction and inflammation cluster included CSF Ng, VILIP1, SNAP25, YKL-40 and sTREM2 (see Fig. 3). The MMSE, CSF pT175/T175 and PRS did not fit into any of the main clusters. The three non-core Alzheimer’s disease pathology clusters also demonstrated strong associations within their respective clusters, although some associations were observed between neuronal dysfunction and inflammation markers and other clusters (see Fig. 2 and Supplementary Fig. 8 for a ShinyApp of individual associations by group and threshold).

There were several sub-clusters within the four primary clusters defined in the entire cohort. Within the core Alzheimer’s disease pathology cluster, amyloid PET associated with CSF A*β*42/40_lumi_ and several CSF phosphorylated tau sites including pS208/S208, pT231/T231, pT153/T153, pT111/T111 and pT217/T217 (see Figs 1–3). Plasma A*β*42/40_plasma_ was also within the core Alzheimer’s disease pathology cluster and was most similar to tau PET and CSF pT208/T208 but was less strongly related to other markers of amyloidosis. Tau PET and the CDR-SB were highly related to one another and to a lesser extent with other amyloid markers. The Alzheimer’s disease cortical signature from the neurodegeneration cluster and CSF pT205/T205 from the AT8-associated tau phosphorylated sites cluster were close in proximity but were not initially in the same cluster (see Fig. 3).

**Figure 4:**
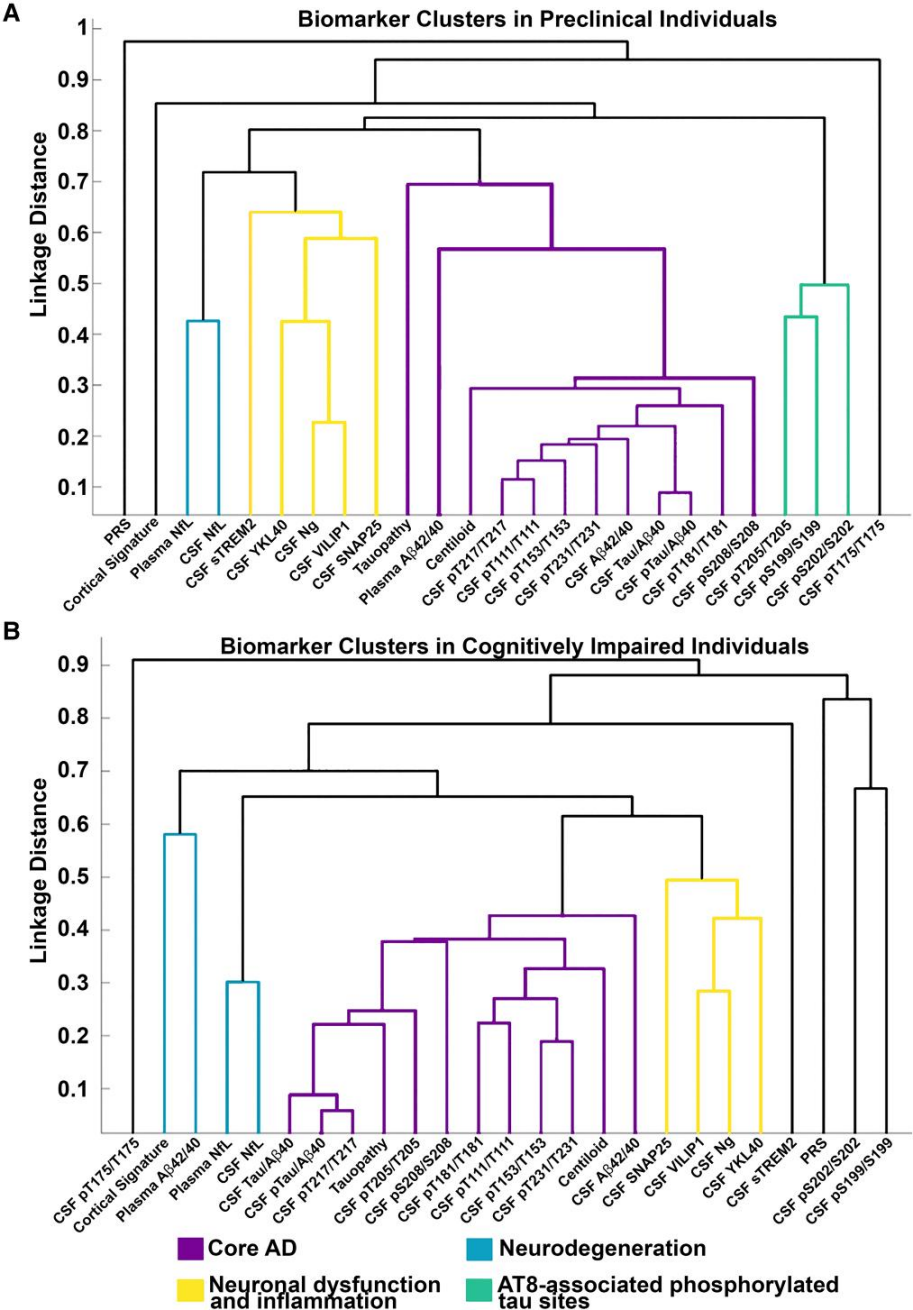
**Agglomerative hierarchical clustering results remain relatively stable across the Alzheimer’s disease trajectory.** (**A**) Biomarker clusters for preclinical Alzheimer’s disease individuals (*n* = 228). In preclinical [cognitively normal (CDR) = 0], amyloid PET-positive individuals), the core Alzheimer’s disease pathology cluster retained all biomarkers that were present for the entire cohort. The AT8-associated phosphorylated tau site and neuronal dysfunction and inflammation clusters similarly retained all markers while the Alzheimer’s disease cortical signature was dropped from the neurodegeneration cluster. (**B**) Biomarker clusters for cognitively impaired individuals (*n* = 88). In cognitively impaired (CDR > 0) individuals, the core Alzheimer’s disease cluster retained all biomarkers (with the exception of A*β*42/40_plasma_) and added pT205/T205. A*β*42/40_plasma_ clustered with the Alzheimer’s disease cortical signature in the neurodegeneration group. Both plasma and CSF NfL grouped together in a separate cluster. The AT8-associated phosphorylated tau site cluster was no longer grouped with other biomarkers. The *y*-axis represents the linkage distance and reflects the absolute value of the pairwise Spearman correlation between biomarkers. Lines are colour coded by cluster. CDR, Clinical Dementia Rating Scale; MMSE, Mini-Mental State Examination; NfL, neurofilament light; Ng, neurogranin; PRS, polygenic risk score; SNAP-25, synaptosomal-associated protein-25; sTREM2, soluble triggering receptor expressed on myeloid cells 2; VILIP-1, visinin-like protein 1; YKL-40, chitinase-3-like protein 1.

As shown in Fig. 4, associations within the core Alzheimer’s disease pathology cluster remained relatively stable across the disease trajectory (i.e. in preclinical and cognitive impairment stages), while the neurodegeneration, AT8-associated tau phosphorylation and neuronal dysfunction and inflammation clusters demonstrated shifts, especially later in the disease course. Within preclinical-only individuals, all clusters retained their respective markers except for the neurodegeneration cluster, which no longer included the Alzheimer’s disease cortical signature (see Fig. 4A). These results were similar to those observed for all amyloid PET-positive individuals, regardless of cognitive status (see Supplementary Fig. 9). When assessing cognitively impaired individuals, the core Alzheimer’s disease pathology cluster retained all biomarkers (with the exception of A*β*42/40_plasma_) and also included the CSF phosphorylated tau site pT205/T205. For this group, A*β*42/40_plasma_ clustered with the Alzheimer’s disease cortical signature. The CSF pS199/S199 and pS202/S202 from the AT8-associated sites did not cluster with any biomarkers (see Fig. 4B).

### Amyloid status feature selection

**Figure 5:**
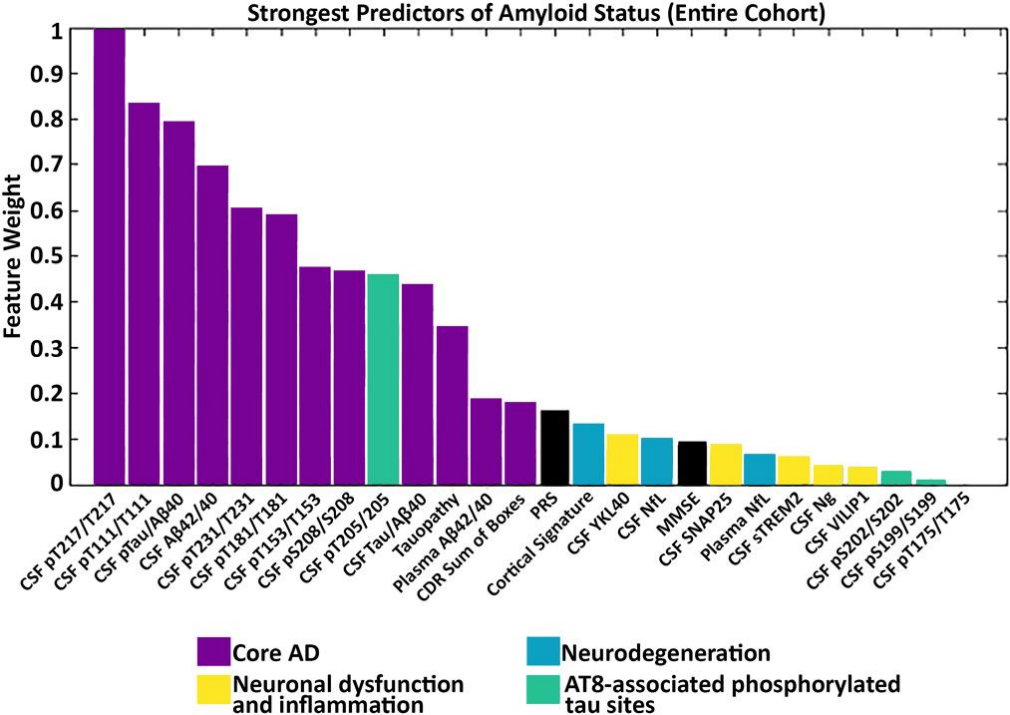
**Random forest feature selection identifies core Alzheimer’s disease pathology markers as the strongest predictors of amyloid status across all individuals.** The strongest predictors of amyloid-positive status were pT217/T217, pT111/T111, p-tau/A*β*40_lumi_ and CSF A*β*42/40_lumi_. Bars are colour coded by cluster. CDR, Clinical Dementia Rating Scale; MMSE, Mini-Mental State Examination; NfL, neurofilament light; Ng, neurogranin; PRS, polygenic risk score; SNAP-25, synaptosomal-associated protein-25; sTREM2, soluble triggering receptor expressed on myeloid cells 2; VILIP-1, visinin-like protein 1; YKL-40, chitinase-3-like protein 1.

Across the entire cohort, without stratifying by cognitive or amyloid status, core Alzheimer’s disease pathology markers were the strongest predictors of amyloid PET status (see Fig. 5). Specifically, CSF pT217/T217 was the strongest predictor of amyloid PET status followed by CSF pT111/T111 and CSF p-tau/A*β*40_lumi_. Other good predictors of amyloid status (i.e. predictor strength >0.5) included CSF A*β*42/40_lumi_, pT231/T231 and pT181/T181. The remaining core Alzheimer’s disease pathology markers, including plasma A*β*42/40_plasma_ and PET tauopathy, as well as measures within neurodegeneration and neuronal dysfunction and inflammation clusters were weaker predictors of amyloid status compared to the CSF biomarkers.

### Cognitive impairment status feature selection

**Figure 6:**
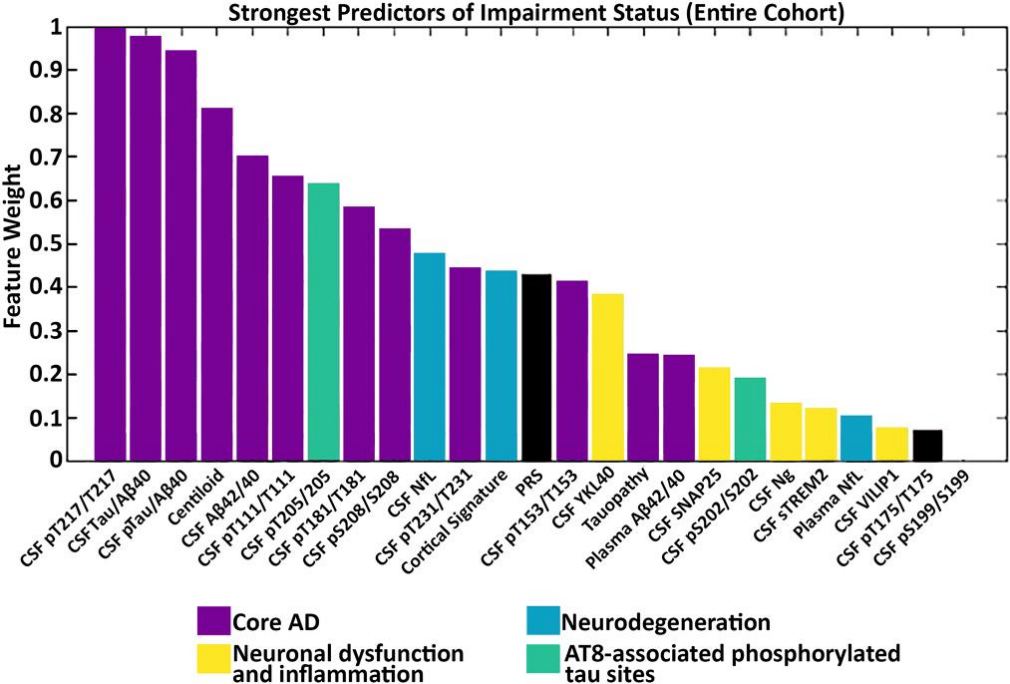
**Random forest feature selection identifies core Alzheimer’s disease pathology markers as the strongest predictors of cognitive impairment across all individuals.** The strongest predictors of cognitive impairment were pT217/T217, t-tau/A*β*40_lumi_, p-tau/A*β*40_lumi_ and amyloid PET. Bars are colour coded by cluster. CDR, Clinical Dementia Rating Scale; MMSE, Mini-Mental State Examination; NfL, neurofilament light; Ng, neurogranin; PRS, polygenic risk score; SNAP-25, synaptosomal-associated protein-25; sTREM2, soluble triggering receptor expressed on myeloid cells 2; VILIP-1, visinin-like protein 1; YKL-40, chitinase-3-like protein 1.

Feature selection demonstrated that across the entire cohort, core Alzheimer’s disease pathology biomarkers were also the strongest predictors of cognitive impairment. CSF pT217/T217 was the strongest predictor followed by CSF t-tau/A*β*40_lumi_ and CSF p-tau/A*β*40_lumi_. Several other biomarkers also had good predictor strength (>0.5) for cognitive status including amyloid PET, CSF A*β*42/40_lumi_, pT111/T111, pT205/T205, pT181/T181 and pS208/S208. In the context of data on all other biomarkers, measures that did not strongly predict cognitive impairment in the random forest model included PET tauopathy, CSF measures of neurodegeneration, neuronal dysfunction and inflammation, as well as A*β*42/40_plasma_. Full feature selection results are shown in Fig. 6.

**Figure 7:**
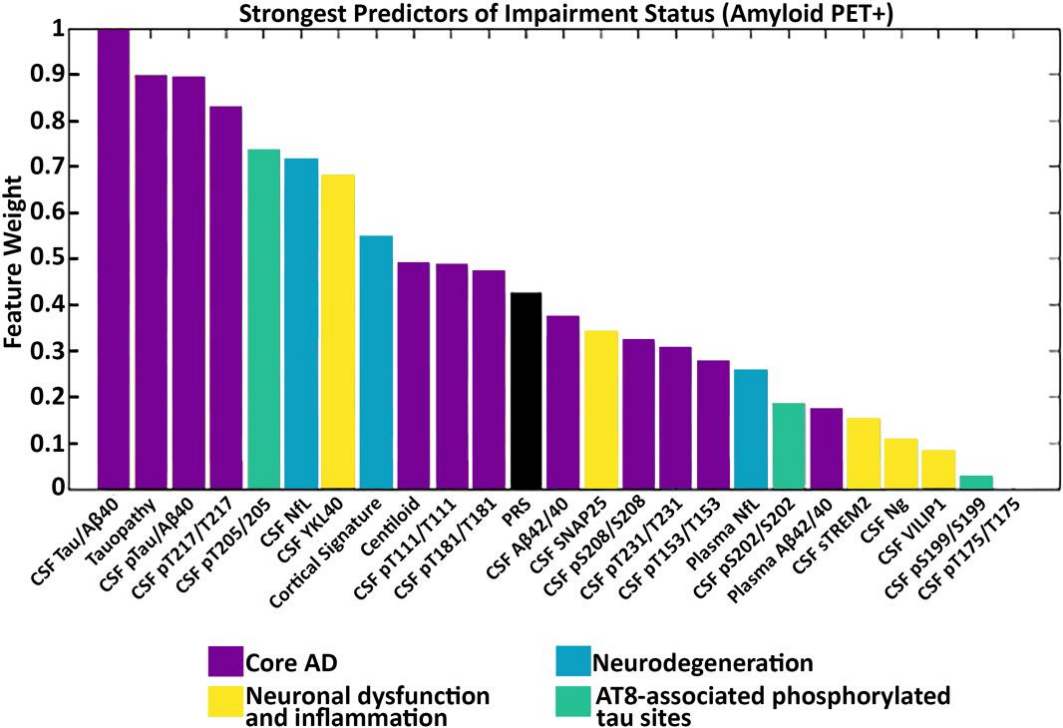
**Random forest feature selection identifies core Alzheimer’s disease pathology markers as the strongest predictors of cognitive impairment in amyloid PET-positive individuals.** The strongest predictors of cognitive impairment were t-tau/A*β*40_lumi_, tau PET, p-tauA*β*40_lumi_ and pT217/T217. Bars are colour coded by cluster. CDR, Clinical Dementia Rating Scale; MMSE, Mini-Mental State Examination; NfL, neurofilament light; Ng, neurogranin; PRS, polygenic risk score; SNAP-25, synaptosomal-associated protein-25; sTREM2, soluble triggering receptor expressed on myeloid cells 2; VILIP-1, visinin-like protein 1; YKL-40, chitinase-3-like protein 1.

When restricting to only amyloid PET-positive individuals, core Alzheimer’s disease pathology markers were still the strongest predictors of cognitive impairment. CSF t-tau/A*β*40_lumi_ was the strongest predictor followed by tau PET, CSF p-tau/A*β*42_lumi_, pT217/T217 and pT205/T205 and downstream markers (CSF NfL, YKL-40, cortical signature; see Fig. 7). Other markers from the core Alzheimer’s disease pathology group including amyloid PET, CSF pT111/T111, CSF pT181/T181 and CSF A*β*42/40_lumi_ as well as the PRS were only modest predictors of cognitive impairment in amyloid PET-positive individuals. The remaining measures of neuronal dysfunction and inflammation, as well as the AT8-associated phosphorylated tau sites, were poor predictors of cognitive impairment.

## Discussion

While an increasing richness of biomarker data has become available to the field, these data are rarely integrated into a joint model to understand the Alzheimer’s disease biological cascade. The present study determined the interrelationships and utility of 27 neuroimaging and fluid biomarkers available for Alzheimer’s disease research. Overall, four primary biomarker clusters emerged: core Alzheimer’s disease pathology, neurodegeneration, AT8-associated tau phosphorylation and neuronal dysfunction and inflammation. These clusters remained relatively stable throughout the disease course, albeit with some subtle shifts in the non-core Alzheimer’s disease pathology clusters. Of the 27 biomarkers assessed, a select few demonstrated strong predictive abilities for each stage of the disease. Markers with the greatest utility were consistently from the core Alzheimer’s disease pathology cluster with minimal additive value coming from the other clusters. Collectively, these results indicate that from a wide array of biomarkers commonly used in Alzheimer’s disease research, many capture shared variance indicating a relatively low level of biological dimensionality. Further, only a handful of biomarkers may provide meaningful utility.

The core Alzheimer’s disease pathology group, as defined from the entire cohort, included neuroimaging and fluid biomarkers of amyloid, most of the CSF tau measures (with the exception of pT205/T205, pT175/T175, pS199/S199 and pS202/S202) and CDR-SB. This cluster had the fewest shifts compared to other clusters across the disease spectrum. In preclinical individuals, all biomarkers in this cluster were retained. In cognitively impaired individuals, all biomarkers in the cluster were retained, with the exception of A*β*42/40_plasma_, while CSF pT205/T205 joined this cluster. A*β*42/40_plasma_ has previously been shown to have the greatest utility early in the disease^54,55^ and is most sensitive to a binary, rather than continuous characterization of amyloid pathology. Therefore, plasma measures may not track with other amyloid measures later in the disease process. In contrast, CSF pT205/T205 has previously been shown to be elevated later in the disease course^10^ and was associated with Alzheimer’s disease core pathology for cognitively impaired individuals.

Feature selection demonstrated that across the entire cohort, core Alzheimer’s disease pathology markers (e.g. CSF measures of pT217/T217, pT111/T111 and p-tau/A*β*40_lumi_) were the strongest predictors of a positive amyloid PET scan. The tight coupling between CSF phosphorylated tau measures, particularly pT217/T217, pT111/T111, pT213/T231 and pT153/T153, and traditional amyloid markers across the disease spectrum supports the changing view in the field that these markers reflect a response to amyloidosis rather than a soluble measure of NFT pathology as measured by tau PET.^1,10,14,56^ Similar results have been seen with other older adult cohorts^11,12^ as well as autosomal dominant Alzheimer’s disease.^57^

When predicting cognitive impairment across the entire cohort, CSF pT217/T217, CSF t-tau/A*β*40_lumi_, CSF p-tau/A*β*40_lumi_ and amyloid PET were the strongest predictors, likely due to their robust ability to identify the presence of underlying Alzheimer’s disease pathophysiology. Tau PET was not a strong predictor, likely because in a general sample increased tau is largely dependent on the presence of abnormal amyloid as measured by PET, CSF and plasma markers also included in the model. However, when restricting predictions to amyloid PET-positive individuals, CSF t-tau/A*β*40_lumi_, tau PET and CSF p-tau/A*β*40_lumi_ were the strongest predictors of cognitive impairment followed by CSF pT217/T217. Tau PET was strongly associated with cognitive status in amyloid PET-positive individuals and its prominence in this model is highly consistent with previous literature.^58,59^ Although typically thought to reflect early disease changes, our models suggest CSF t-tau/A*β*40_lumi_, p-tau/A*β*40_lumi_ and pT217/T217 reflect a degree of increasing disease severity at least through the early stages of cognitive impairment and reflect and intermediate process between early amyloidosis and the formation of NFTs. Further, CSF pT217/T217 is a particularly powerful Alzheimer’s disease biomarker because it reflects both amyloid and tau pathology.

After the core Alzheimer’s disease pathology cluster, markers of neurodegeneration provided the next greatest utility in predicting cognitive status. This cluster demonstrated modest shifts throughout the disease spectrum. Markers of neurodegeneration included CSF and plasma NfL and the Alzheimer’s disease cortical signature within cognitively unimpaired amyloid positive whereas, in symptomatic Alzheimer’s disease, plasma A*β*42/40 was also included. This cluster likely represents later downstream Alzheimer’s disease-dependent neurodegenerative processes and was typically the cluster most closely related to the core Alzheimer’s disease pathology biomarkers.

A subset of the CSF tau phosphorylation sites associated with the AT8 epitope^60^ (pS202/S202, pS199/S199, pT175/T175) clustered together but had relatively poor predictive performance. Although some of these measures were associated with neurodegeneration and Alzheimer’s disease core pathology clusters within the general cohort, they were relatively non-specific and were excluded from the clusters in symptomatic individuals. Research from our group has previously demonstrated that these sites are less likely to be phosphorylated compared to other tau sites and are inversely associated with amyloid PET.^10^ Collectively, these tau phosphorylation sites have a lack of specificity for Alzheimer’s disease pathology.

Finally, markers of neuronal dysfunction and inflammation were highly clustered with one another and were separate from other clusters. Although these markers are thought to reflect different biological properties (e.g. inflammation, neuronal death, pre- and post-synaptic dysfunction), it is possible that they represent processes that are so inherently interrelated that the information provided by any measure is largely redundant. Alternatively, there may be some structured variance (i.e. overall fluctuations in CSF protein levels), which overshadows any disease effects seen with these biomarkers. Outside of CSF YKL-40, the neuronal dysfunction and inflammation measures also had very little relationship with either amyloid or cognitive status. As a result, the majority of these markers (CSF Ng, VILIP-1, sTREM2, SNAP-25) are likely to provide minimal utility in the context of Alzheimer’s disease.

While the current study provides a comprehensive phenotyping of commonly used biomarkers, several limitations should be noted. The Knight ADRC places a strong emphasis on recruiting preclinical individuals with the aim of understanding the transition to dementia. As a result, the current cohort includes a modest population of impaired individuals that are largely in the early symptomatic stages of the disease. Further, this cohort overwhelmingly represents individuals with typical amnestic phenotypes as all cognitively impaired, amyloid PET-negative individuals were removed from the analyses. Therefore, it is unclear whether similar results would be obtained in cohorts with more severe dementia and more varied presentations, as well as non-Alzheimer’s disease dementias.

Although the current work considers a breadth of different biomarkers, only two plasma markers (NfL_plasma_ and A*β*42/40_plasma_) were included. As plasma measures become more widely available, it will be informative to incorporate such markers into similar analyses to determine their utility, particularly relative to their CSF counterparts. Similarly, while our study utilized PET to determine amyloid positivity, future analyses should compare results with those yielded from additional fluid cut-offs. While being the most comprehensive simultaneous examination of biomarkers to date, some data for some biomarkers was not available for the cohort, particularly measures of microtubule binding region (MTBR)^61^ and glial fibrillary acidic protein (GFAP).^62^ Both markers have recently demonstrated a high utility in measuring tau and inflammation, respectively. Further, the current work examines cross-sectional associations. Future studies would benefit from the assessment of longitudinal trajectories.

Lastly, the current work utilizes ML to simultaneously understand the structure and utility of a very large number of different Alzheimer’s disease biomarkers. Prior work in the literature has typically focused on pairwise correlations between makers^8,10-12,14,63^ or assessed the utility of only a handful of markers in predicting biomarker and clinical outcomes using linear regression or classification (e.g. AUC) approaches.^8,10,11,13,14,64-67^ ML approaches are uniquely suited for large, high- dimensional datasets with potential nonlinear associations whereas more restricted models are strongest at testing a focused hypothesis. ML also represents a general approach to holistically analysing data rather than using one specific model. The utilization of a random forest was chosen because of its ability to handle missing values. This ensured we were able to preserve as much data as possible for training, which is essential for ML efficacy. Nevertheless, missing values may have influenced results. As more comprehensive datasets emerge, future work should validate the present findings. Alternatively, strategies like data imputation and other ML approaches could be employed.

## Conclusion

As the number of biomarkers used in Alzheimer’s disease research continues to grow, it becomes increasingly important to determine which biomarkers provide unique information and which have the greatest utility in predicting clinical outcomes. The present study identified four main clusters of biomarkers: core Alzheimer’s disease pathology, neurodegeneration, AT8-associated tau phosphorylation and neuronal dysfunction and inflammation. Rather than being measures of NFT, the clustering and predictive models demonstrate that biomarkers of phosphorylated tau measured using immunoassays or mass spectrometry reflect a response to amyloid plaques. Notably, CSF pT217/T217, along with tau PET, had high predictive value in identifying individuals with cognitive impairment even after the onset of abnormal amyloid accumulation. MRI, CSF, plasma measures of NfL, and CSF YKL-40 had a moderate ability to predict impairment. The majority of emerging biomarkers added little to the prediction of cognitive impairment in the context of other Alzheimer’s disease biomarker data. Overall, this work suggests that of the many biomarkers used in Alzheimer’s disease research, only a handful have meaningful clinical utility.

## Supplementary Material

fcae081_Supplementary_Data

## Data Availability

Data associated with this study are available in the main text or the Supplementary material and can be shared with external investigators upon submission of a proposal and under a data-user agreement.
