# Supplementary material for: Comparison of cerebrospinal fluid, plasma and neuroimaging biomarker utility in Alzheimer’s disease
Source: Brain Commun. 2024 Mar 15;6(2):fcae081. doi: 10.1093/braincomms/fcae081 (PMC10950051; doi:10.1093/braincomms/fcae081)
Supplement: fcae081_Supplementary_Data [file fcae081_supplementary_data.docx]

**Supplementary Materials**

**S1. Methods**

**S1.1 Magnetic Resonance Imaging**

Imaging was performed using either a 3.0 Tesla Siemens Biograph mMR (Erlangen, Germany) or 3.0 Tesla Siemens TIM Trio (Erlangen, Germany) scanner. For the Siemens Biograph mMR, high-resolution 3-dimensional sagittal T1 magnetization prepared – rapid gradient echo (MP-RAGE) anatomical images were acquired with the scanning parameters of repetition time (TR) = 2300 ms, time to echo (TE) = 2.95 ms, flip angle = 9°, 176 slices, acquisition matrix = 240 x 256, and voxel size = 1 x 1 x 1.2 mm^3^. For the Siemens TIM Trio, high-resolution 3-dimensional sagittal T1 MP-RAGE anatomical images were acquired with the scanning parameters of TR = 2400 ms, TE = 3.16 ms, flip angle = 8°, 176 slices, acquisition matrix = 256 x 256, and voxel size = 1 x 1 x 1 mm^3^.

**S1.2 Positron Emission Tomography Imaging**

Participants received a single intravenous bolus of PiB or ^18^ F-AV-45 infused for 20 seconds. Three-dimensional axial PET images were acquired with the scanning parameters of 176 slices, acquisition matrix = 256 x 256, and voxel size = 1.12 x 1.12 x 2.03 mm^3^. Attenuation correction was performed using computed tomography (CT). An in-house PET unified pipeline (PUP) was used to process PET images^2,3^. Partial volume correction was performed with a regional spread function (RSF), which accounts for signal spillover from non-regions of interest and nonbrain regions^3^. To standardize across PiB and ^18^ F-AV-45, standard uptake value ratios (SUVRs) were converted to Centiloids^4,5^. Briefly, the Centiloid scale is defined by two anchor points: the mean amyloid burden of a young control group, and the mean amyloid burden of an Alzheimer disease (AD) group. The mean amyloid burden of the AD group was represented as 100 in the Centiloid scale. Regression and linear transformation were performed to calibrate the tracers and local processing methods to the Centiloid scale^4^.

**Supplementary Table 1**

| Amyloid PET | CSF pT205/T205 |
| --- | --- |
| Tau PET | CSF pS208/S208 |
| MRI Cortical Signature | CSF pT217/T217 |
| Plasma A*β*42/40 | CSF pT231/T231 |
| Plasma NfL | CSF NfL |
| CSF A*β*42/40_lumi_ | CSF Ng |
| CSF p-tau/A*β*40_lumi_ | CSF SNAP-25 |
| CSF tau/A*β*40_lumi_ | CSF VILIP-1 |
| CSF pT111/T111 | CSF YKL-40 |
| CSF pT153/T153 | CSF sTREM2 |
| CSF pT175/T175 | CDR Sum of Boxes |
| CSF pT181/T181 | MMSE |
| CSF pS199/S199 | PRS |
| CSF pS202/S202 |  |

Abbreviations: PET = positron emission tomography; MRI = magnetic resonance imaging; CSF = cerebrospinal fluid; NfL = neurofilament light; Ng= neurogranin; SNAP-25 = synaptosomal-associated protein-25; VILIP-1 = visinin-liken protein 1; YKL-40 = Chitinase-3-like protein 1; soluble triggering receptor expressed on myeloid cells 2 (sTREM2); CDR = Clinical Dementia Rating Scale; MMSE = Mini-Mental State Examination; PRS = polygenic risk score.


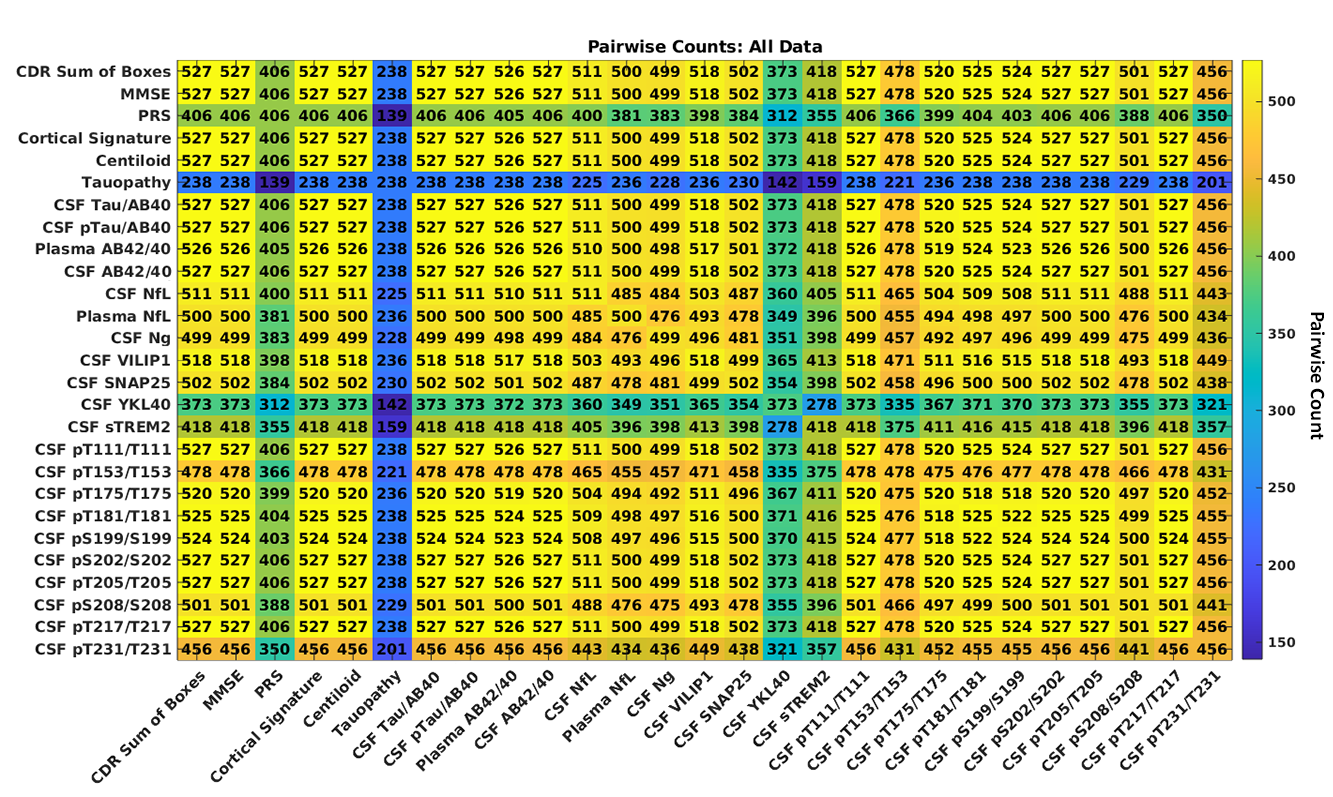


**Supplementary Figure 1.** **Pairwise counts for all biomarkers in the entire cohort (n=527)**. Biomarker pairs with higher counts are shown in warm colors and pairs with lower counts are shown in cool colors.


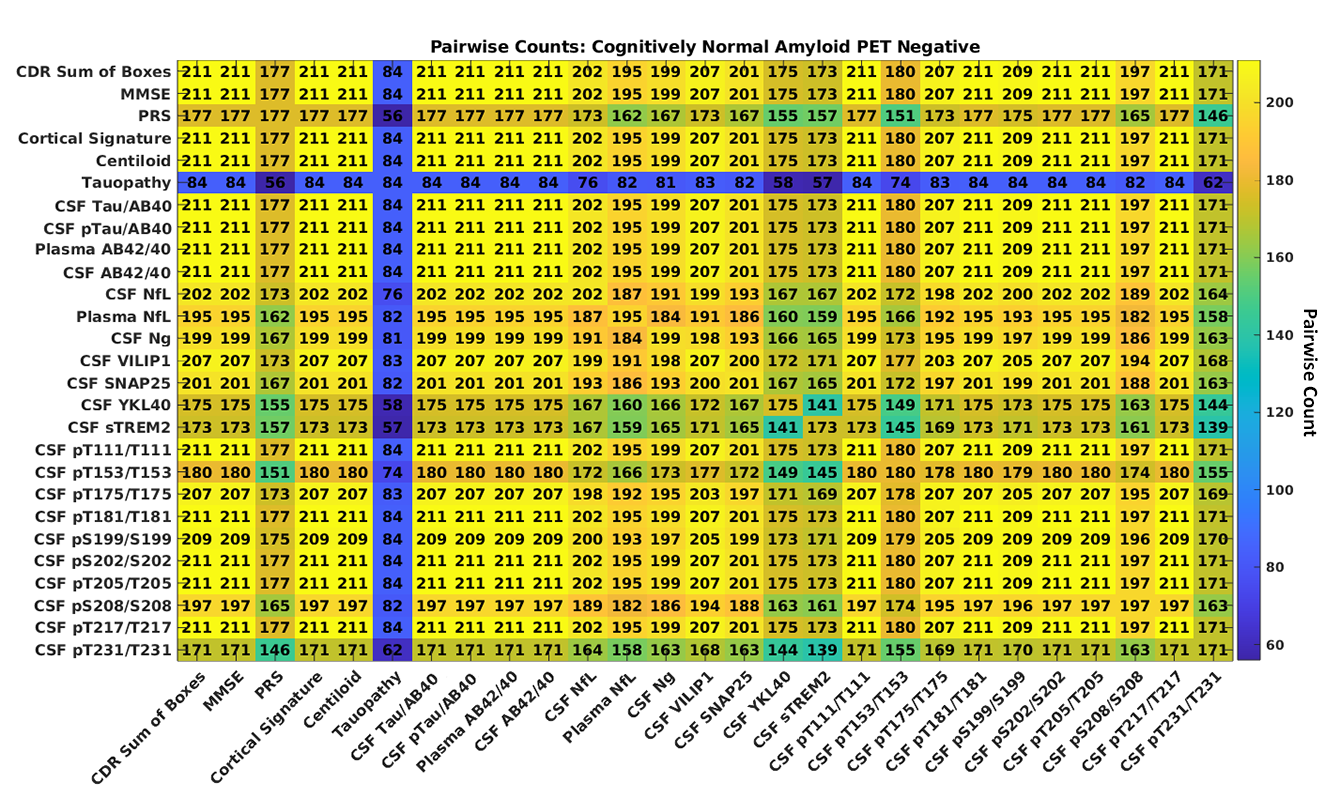


**Supplementary Figure 2.** **Pairwise counts for all biomarkers in cognitively normal, amyloid positron emission tomography (PET) negative individuals (n=211)**. Biomarker pairs with higher counts are shown in warm colors and pairs with lower counts are shown in cool colors.


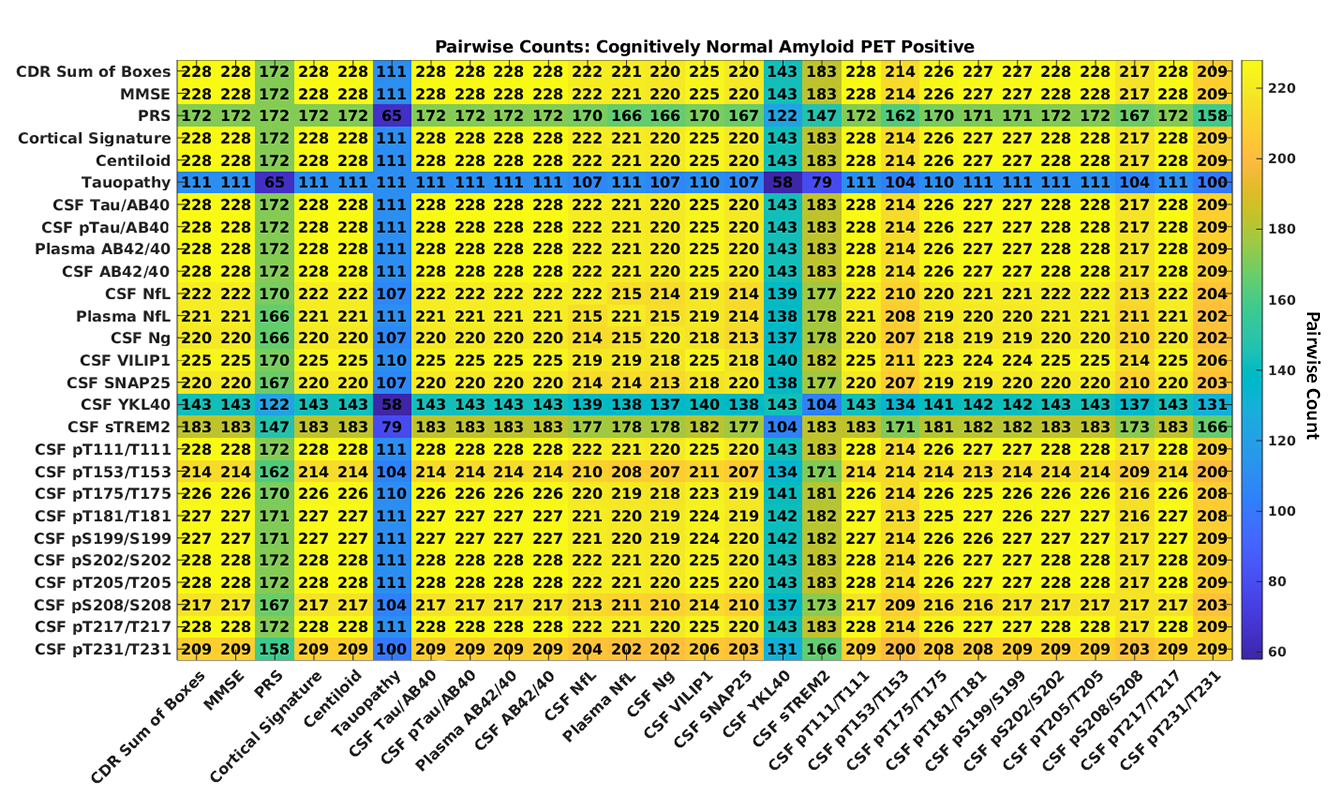


**Supplementary Figure 3.** **Pairwise counts for all biomarkers in cognitively normal, amyloid positron emission tomography (PET) positive individuals (n=228)**. Biomarker pairs with higher counts are shown in warm colors and pairs with lower counts are shown in cool colors.


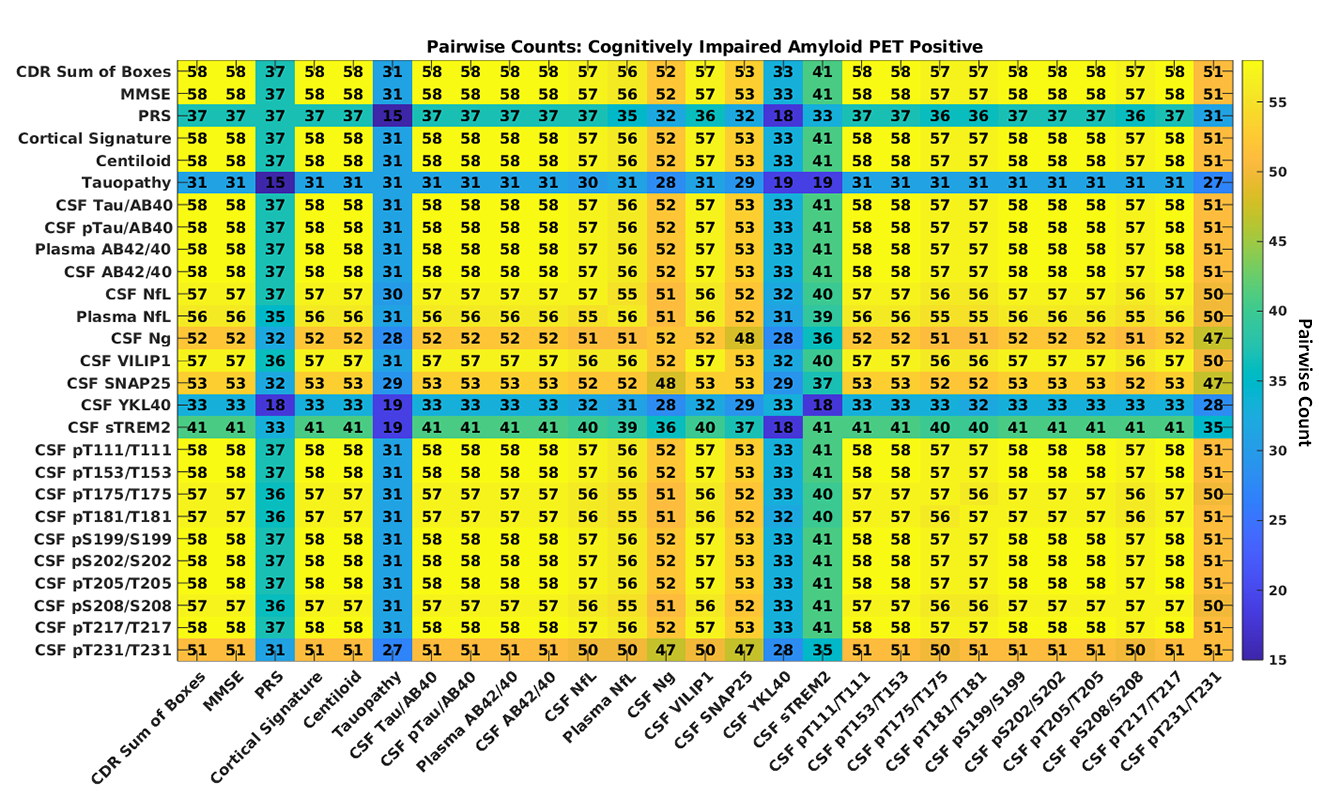


**Supplementary Figure 4.** **Pairwise counts for all biomarkers in cognitively impaired, amyloid positron emission tomography (PET) positive individuals (n=88)**. Biomarker pairs with higher counts are shown in warm colors and pairs with lower counts are shown in cool colors.


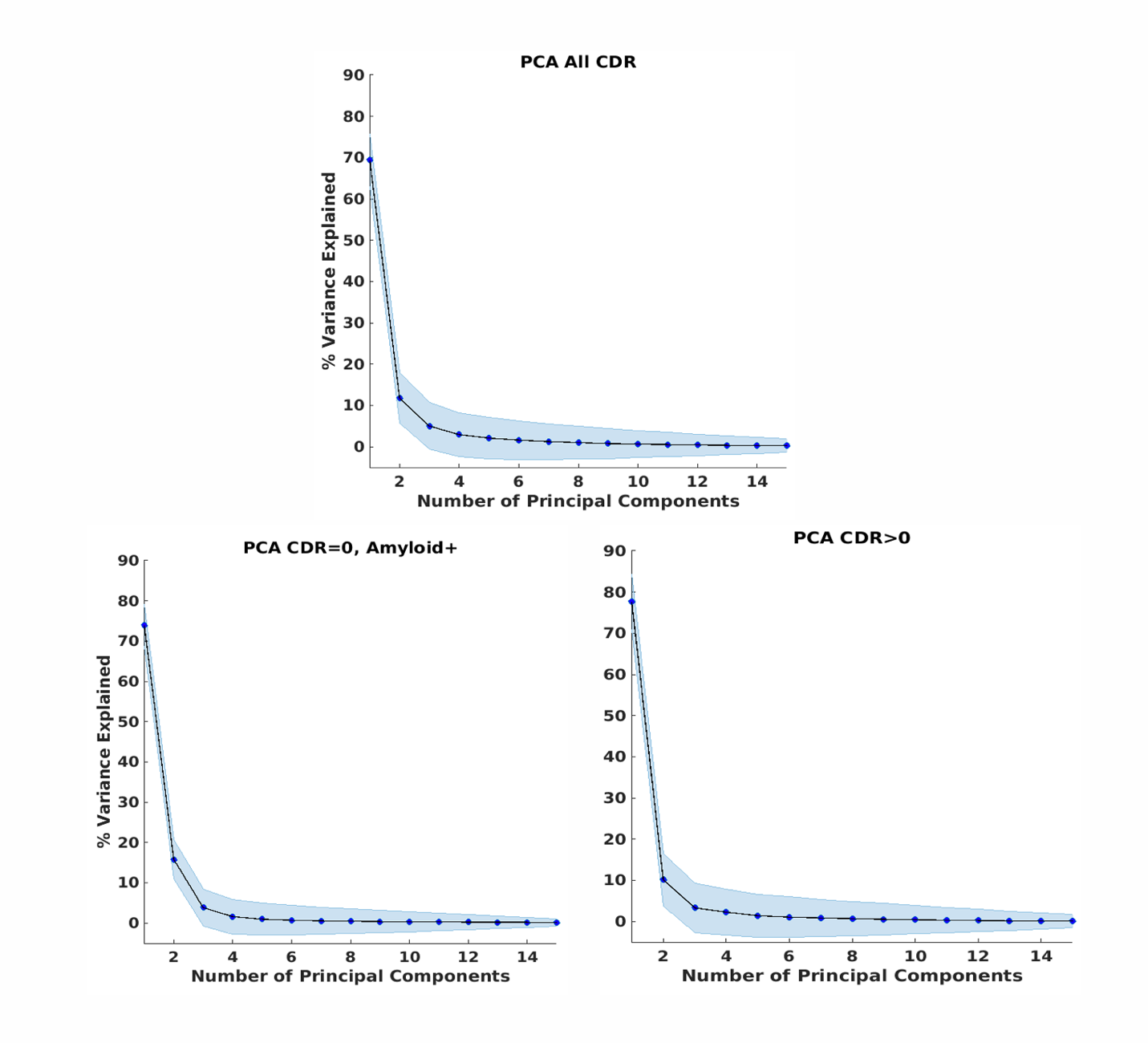
**Supplementary Figure 5. Elbow method for identification of optimal number of clusters**. The optimal number of clusters was identified using the elbow method^6^ based on principal component analysis (PCA). The elbow method relies on identifying cluster solutions based on a minimum amount of explained variance and a point of diminishing return (i.e., at what point does adding an additional cluster no longer provide a meaningful increase in the explained variance). Results indicate that a minimum of 3 to 4 clusters are needed to explain the greatest variance. Abbreviations: CDR = Clinical Dementia Rating Scale.


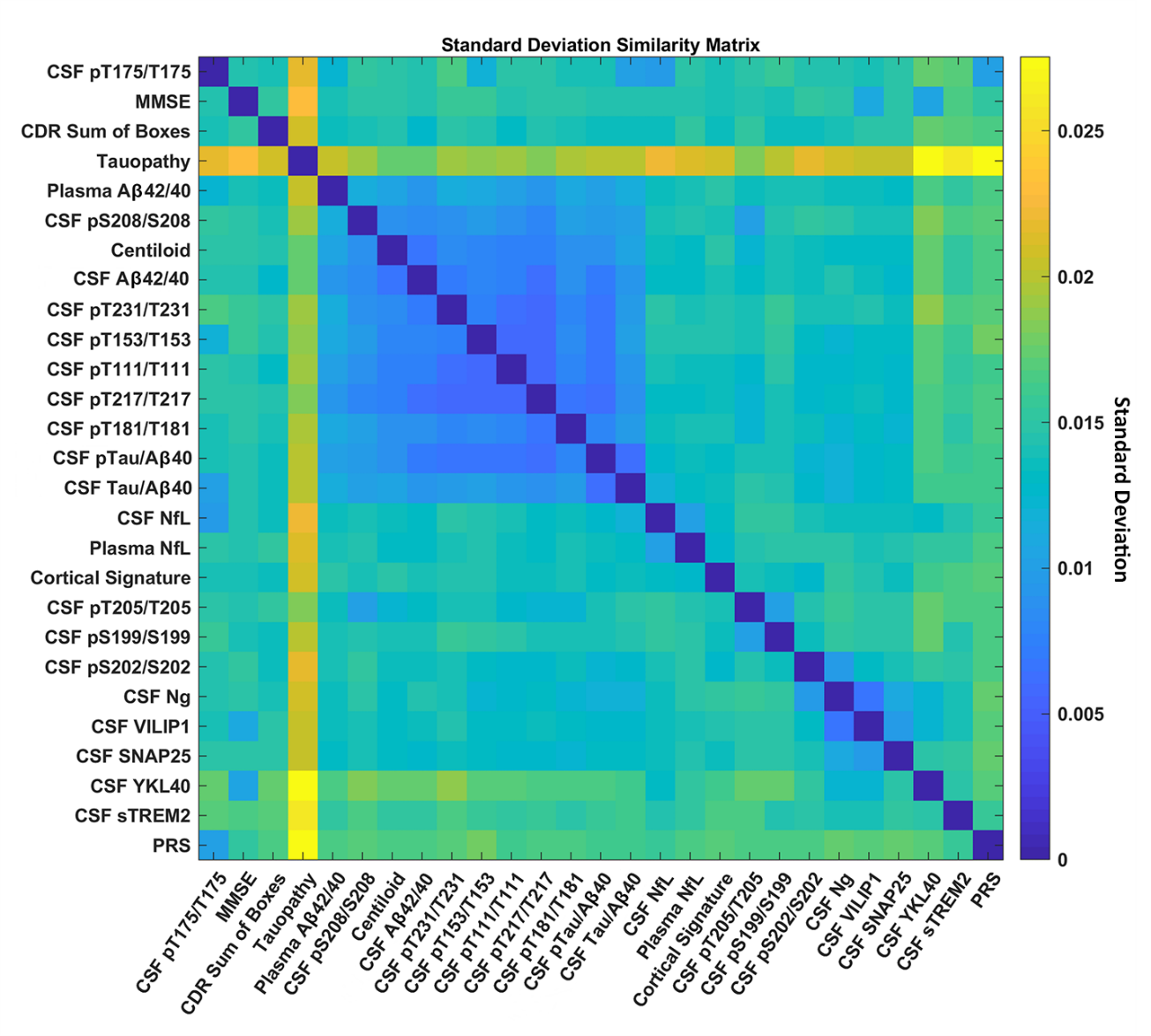


**Supplementary Figure 6. Biomarker standard deviations**. To assess the stability of the results, the principal components were calculated for 1000 random permutations of 80% of the clustering feature space (corresponding to removing 20% of participants from the data set). The mean and standard deviation were then calculated for each analysis. Abbreviations: PET = positron emission tomography; MRI = magnetic resonance imaging; CSF = cerebrospinal fluid; NfL = neurofilament light; Ng= neurogranin; SNAP-25 = synaptosomal-associated protein-25; VILIP-1 = visinin-like protein 1; YKL-40 = Chitinase-3-like protein 1; soluble triggering receptor expressed on myeloid cells 2 (sTREM2); CDR = Clinical Dementia Rating Scale; MMSE = Mini-Mental State Examination; PRS = polygenic risk score.

**S2. Results**


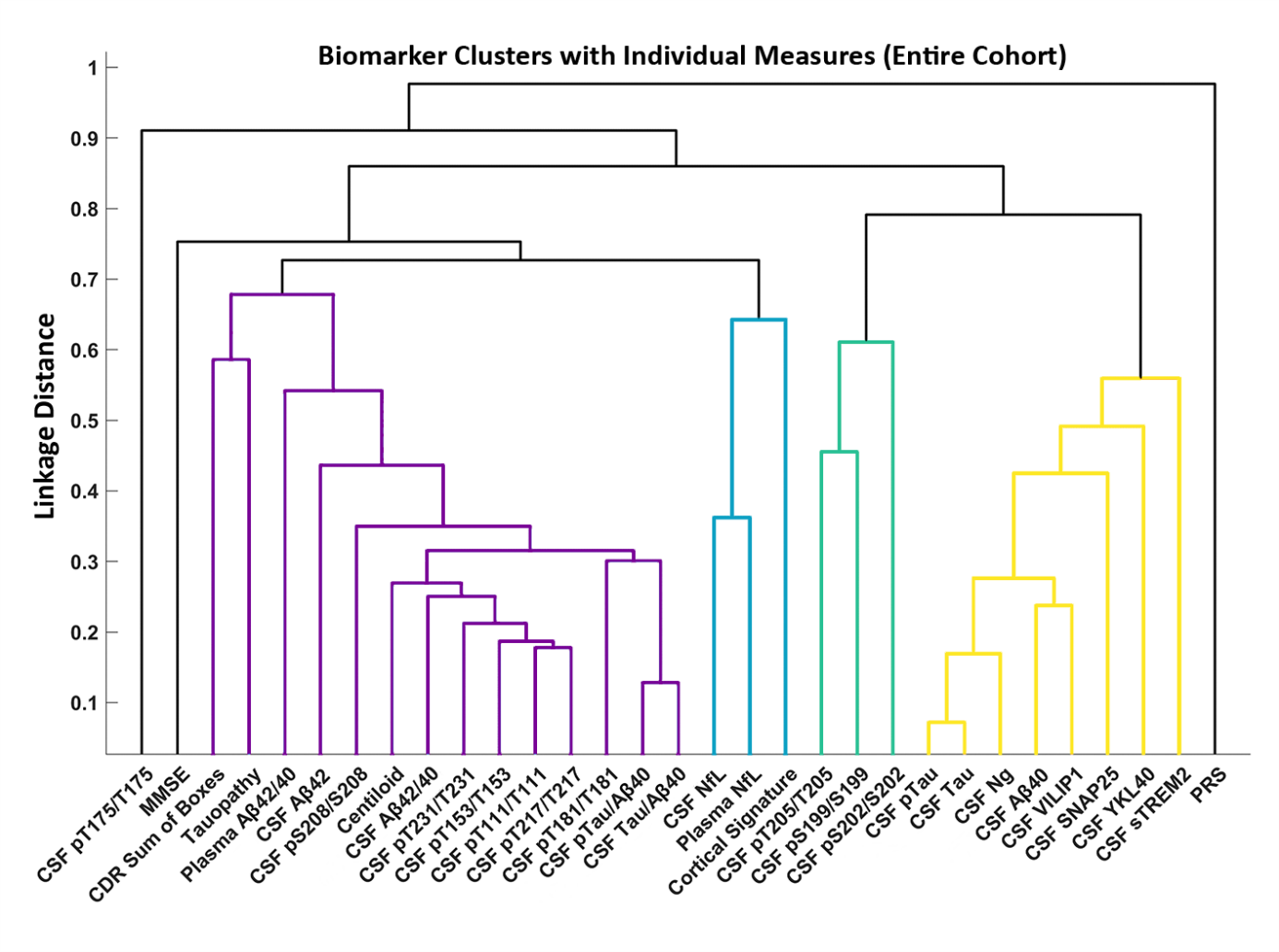
**Supplementary Figure 7.** Hierarchical clustering results with individual measures (i.e., cerebrospinal fluid [CSF] A*β*42, A*β*40, p-tau, and t-tau) added for the entire cohort (n=527). All clusters were the same as those observed in the primary analysis with CSF A*β*42 added to the core AD pathology cluster, and CSF p-tau, t-tau, and A*β*40 added to the neuronal dysfunction and inflammation cluster. The y-axis represents the linkage distance and reflects the absolute value of the pairwise Spearman correlation between biomarkers. Lines are color coded by cluster where purple = core AD, blue = neurodegeneration, green = AT8-associated phosphorylated tau sites, and yellow = neuronal dysfunction and inflammation. Abbreviations: PET = positron emission tomography; MRI = magnetic resonance imaging; NfL = neurofilament light; Ng= neurogranin; SNAP-25 = synaptosomal-associated protein-25; VILIP-1 = visinin-like protein 1; YKL-40 = Chitinase-3-like protein 1; soluble triggering receptor expressed on myeloid cells 2 (sTREM2); CDR = Clinical Dementia Rating Scale; MMSE = Mini-Mental State Examination; PRS = polygenic risk score.

**Supplementary Figure 8.** Circle plots by group and threshold are represented in a ShinyApp and can be found at the following link: <https://dianahobbs.shinyapps.io/CirclePlots/>.


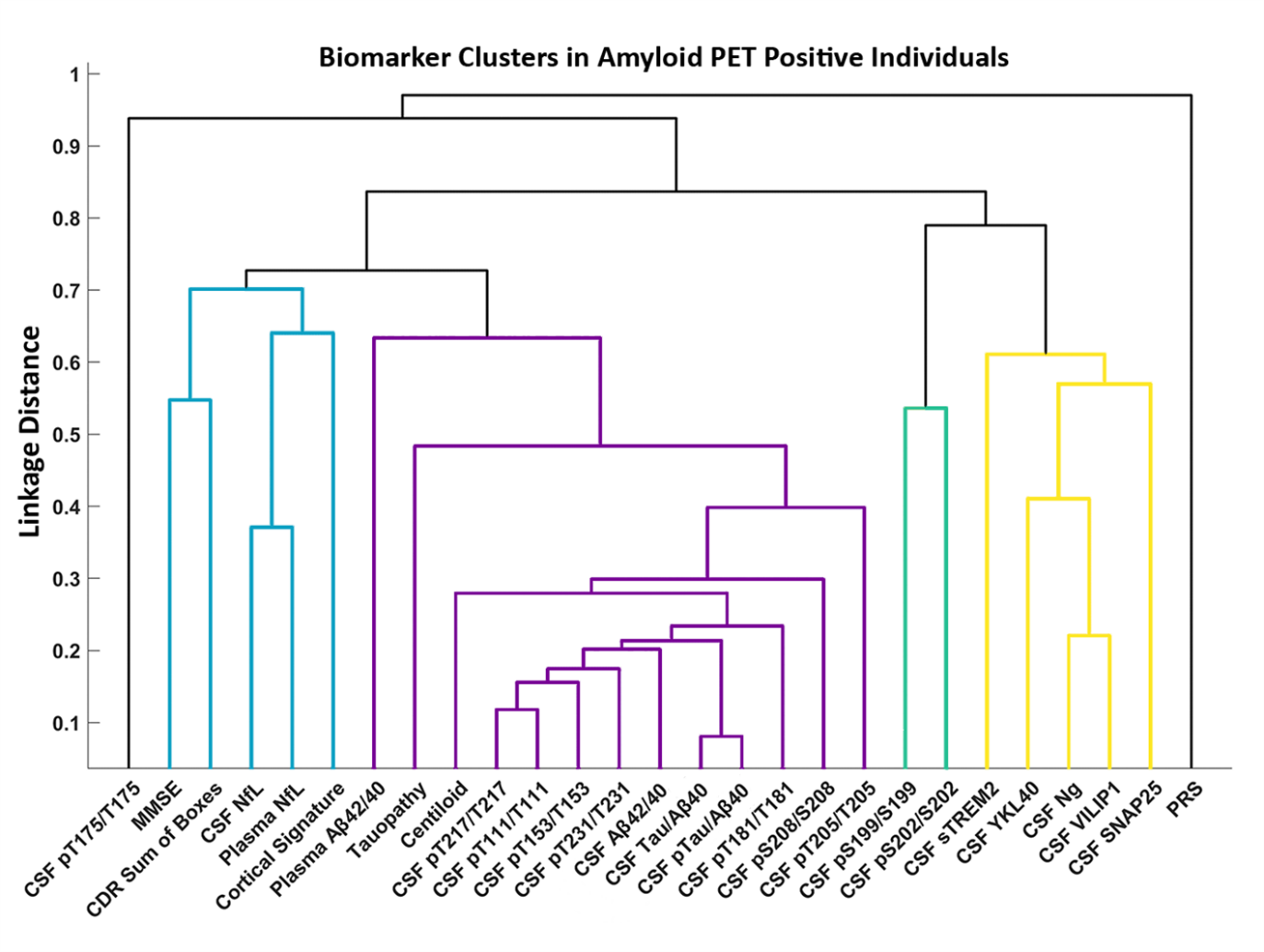


**Supplementary Figure 9.** Hierarchical clustering results in individuals with abnormal amyloid positron emission tomography (PET), regardless of cognitive status (n=316). The core AD cluster retained all biomarkers (with the exception of plasma A*β*42/40) and added phosphorylated tau site 205. Plasma A*β*42/40 clustered with the fluid neurofilament light (NfL) measures and cortical signature in the neurodegeneration group, as did the Clinical Dementia Rating (CDR) Scale Sum of Boxes, the Mini-Mental State Examination (MMSE) and pT175/T175. The y-axis represents the linkage distance and reflects the absolute value of the pairwise Spearman correlation between biomarkers. Lines are color coded by cluster where purple = core AD, blue = neurodegeneration, green = AT8-associated phosphorylated tau sites, and yellow = neuronal dysfunction and inflammation. Abbreviations: MRI = magnetic resonance imaging; CSF = cerebrospinal fluid; Ng= neurogranin; SNAP-25 = synaptosomal-associated protein-25; VILIP-1 = visinin-like protein 1; YKL-40 = Chitinase-3-like protein 1; soluble triggering receptor expressed on myeloid cells 2 (sTREM2); PRS = polygenic risk score.

**References**

1. Cruchaga C, Kauwe JSK, Harari O, et al. GWAS of cerebrospinal fluid tau levels identifies risk variants for alzheimer’s disease. *Neuron*. 2013;78(2):256-268.

2. Su Y, D’Angelo GM, Vlassenko AG, et al. Quantitative Analysis of PiB-PET with FreeSurfer ROIs. Chen K, ed. *PLoS One*. 2013;8(11):e73377. http://dx.plos.org/10.1371/journal.pone.0073377. Accessed February 18, 2020.

3. Su Y, Blazey TM, Snyder AZ, et al. Partial volume correction in quantitative amyloid imaging. 2014. http://dx.doi.org/10.1016/j.neuroimage.2014.11.058. Accessed June 24, 2022.

4. Klunk WE, Koeppe RA, Price JC, et al. The Centiloid project: Standardizing quantitative amyloid plaque estimation by PET. *Alzheimer’s Dement*. 2015;11(1):1-15.e4. https://pubmed.ncbi.nlm.nih.gov/25443857/. Accessed October 7, 2020.

5. Su Y, Flores S, Hornbeck RC, et al. Utilizing the Centiloid scale in cross-sectional and longitudinal PiB PET studies. *NeuroImage Clin*. 2018;19:406-416. https://doi.org/10.1016/j.nicl.2018.04.022. Accessed October 7, 2020.

6. Nainggolan R, Perangin-angin R, Simarmata E, et al. Improved the performance of the K-means cluster using the sum of squared error (SSE) optimized by using the elbow method. In: *Journal of Physics: Conference Series*. Vol 1361. IOP Publishing; 2019:12015.
